# Exploring the Possible Impact of Echocardiographic Diastolic Function Parameters on Outcome in Paediatric Dilated Cardiomyopathy

**DOI:** 10.3390/children9101500

**Published:** 2022-09-30

**Authors:** Sabrina Bressieux-Degueldre, Matthew Fenton, Troy Dominguez, Michael Burch

**Affiliations:** 1 Paediatric Cardiology Department, Great Ormond Street Hospital for Children, London WC1N 3JH, UK; 2 Cardiac Intensive Care Unit, Great Ormond Street Hospital for Children, London WC1N 3JH, UK

**Keywords:** diastolic function, dilated cardiomyopathy, children, echocardiography

## Abstract

Diastolic dysfunction is an important determinant for prognosis and survival in several paediatric heart diseases. We aimed to explore its possible impact on outcome in children with dilated cardiomyopathy. From 2006 to 2016, children less than 18 years old with dilated cardiomyopathy were retrospectively enrolled. Echocardiographic diastolic function parameters and child outcomes were analysed. Of 43 children aged 0.2 to 16.1 years old referred with dilated cardiomyopathy, 8 patients required cardiac transplant or mechanical assist devices (18%), 24 had persistently abnormal left ventricular function and/or dilatation (56%) and 11 patients recovered (26%). There was no significant difference in mitral velocities on Tissue Doppler Imaging, mitral valve inflow velocities, isovolumic relaxation time, left atrial area z-score and mitral lateral E/e’ ratios between patients with recovery and patients with disease progression or persistently abnormal ventricular function and/or dilation. This is the first study on childhood dilated cardiomyopathy to address individual echocardiographic diastolic function parameters and their association to recovery. In this study, echocardiographic parameters for diastolic function did not predict recovery.

## 1. Introduction

Paediatric dilated cardiomyopathy has a poor prognosis and it remains the main indication for paediatric heart transplantation [1]. A multicentre study of new-onset heart failure due to muscle heart disease in the United Kingdom and Ireland found that a third of children died or needed heart transplantation within one year from presentation [2]. One important problem in paediatric dilated cardiomyopathy is the unpredictability of the disease, ranging from spontaneous recovery to intractable heart failure [1,3,4]. This makes family counselling, monitoring and decision making for timing and type of intervention, especially transplant listing, challenging.

Paediatric studies have often focused on systolic function and on left ventricular dilatation as outcome predictors in dilated cardiomyopathy [5,6,7]. Novel laboratory biomarkers are gaining interest in paediatric heart failure [8]. Diastolic function is important in the prognostication of dilated cardiomyopathy in adults [9,10,11] but there is little work in paediatric dilated cardiomyopathy. Diastolic dysfunction, however, is thought to be an important determinant for prognosis and survival in several paediatric heart diseases, such as hypertrophic cardiomyopathy and congenital aortic valve disease [12,13,14], and is part of routine echocardiographic assessment.

The aim of this study was to explore the possible prognostic value of echocardiographic diastolic function parameters in paediatric dilated cardiomyopathy.

## 2. Materials and Methods

We retrospectively reviewed the first echocardiogram in which diastolic function had been assessed in all children aged ≤ 18 years with dilated cardiomyopathy due to myocarditis, familial or idiopathic dilated cardiomyopathy who were referred to our unit between January 2006 and August 2016 and who last had an assessment for outcome between June 2015 and December 2016.

Patients with dilated cardiomyopathy due to other causes, such as ischemic, toxic, neuromuscular, endocrine and metabolic diseases, were excluded. Other forms of cardiomyopathy, for example, mixed-type cardiomyopathy, restrictive and non-compaction cardiomyopathy, were excluded.

Diagnosis of dilated cardiomyopathy was based on two-dimensional echocardiographic criteria with a left ventricular end-diastolic dimension (LVEDD) > 5.5 cm (or z-score > +2 for age) and a left ventricular fractional shortening (LV FS) < 28%.

Outcome was defined as the three following possibilities: recovery, stabilization and progression of heart failure, leading to the need for mechanical circulatory support as bridge-to-transplant, cardiac transplant or death. Recovery was characterized by normalization of left ventricular size and systolic function. Stabilization was defined as persistent impairment of cardiac systolic function and/or persistent LV dilatation. Outcome was assessed at last recorded visit.

For all patients, we recorded age at assessment and timeframe to the last visit. Echocardiographic data, including parameters for LV dimensions (z-scores for LV end-diastolic dimensions and LV end-systolic dimensions in parasternal long axis view), LV systolic function from M-mode in parasternal long axis view and from tissue Doppler Imaging (TDI) in 4-chamber view (LV FS, lateral s’-, septal s’-velocities), were recorded. Echocardiographic parameters for diastolic function included measurements from mitral valve inflow Doppler (mitral A-, mitral E-velocities; mitral E-velocity deceleration time; E/A ratio), measurements from left lateral and septal TDI (lateral e’-, septal e’-, lateral a’-, septal a’-velocities) in 4-chamber view, z-scores for left atrial area (LA area z-score) and isovolumic relaxation time (IVRT). Extracted values of the aforementioned measurements, including mitral E/e’ ratios, were calculated.

The association between individual diastolic and systolic function parameters on initial echocardiogram and outcome at last visit was analysed.

### Statistical Analysis

Baseline characteristics between groups were compared by means of a Kruskal–Wallis test, with the Dunn post-hoc procedure. For further data analysis, patients with persistent impairment of LV dimensions and/or LV systolic function (stabilization) and patients with disease progression (eventually needing cardiac transplant or mechanical assist devices for bridge-to-transplant) were merged and compared to those who recovered.

The Mann–Whitney test was applied to compare continuous data between groups. Categorical data were compared by means of the χ^2^-test. Statistical significance was assigned at *p* < 0.05.

## 3. Results

**Figure 1:**
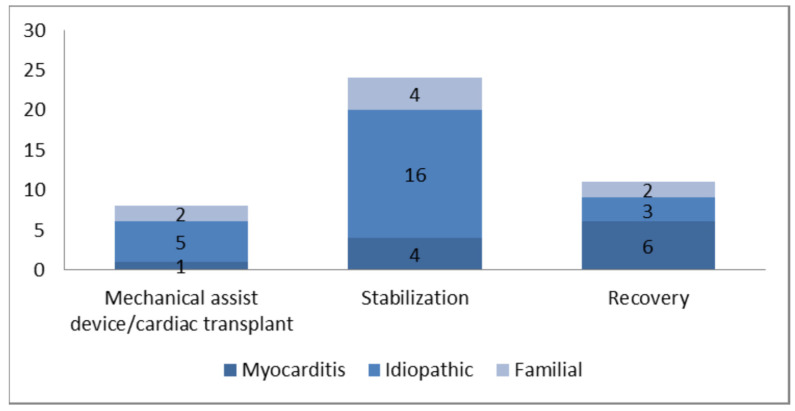
Cause related to outcome.

In total, forty-three baseline echocardiograms of children with dilated cardiomyopathy were reviewed. The cause of dilated cardiomyopathy was familial for 8 patients (18%), idiopathic for 24 (56%) and probable myocarditis for 11 (26%). Of the forty-three children, at the last follow-up, 8 patients had required cardiac transplant or mechanical assist devices for bridge-to-transplant (18%), 24 had persistently abnormal left ventricular function and/or dilatation (56%) and 11 patients had recovered (26%). Probable myocarditis was the most common cause leading to recovery (55%) (Figure 1).

Clinical characteristics are depicted in Table 1. Median age at assessment was 1.8 years (interquartile range, IQR 0.67 to 4.4 years) and median duration of follow up was 3.6 years (IQR 1.4 to 6.0 years). The median duration of follow-up was significantly shorter for patients with disease progression, and they eventually needed an assist device or a heart transplant. No further differences in the baseline characteristics were detected while comparing the three groups.

While looking at LV dimensions and systolic function, results showed that higher left ventricular end diastolic diameter z-scores (LVEDDz) and lower indices for left ventricular systolic function (LV fractional shortening) were predictors for worse outcome (Table 2).

Echocardiographic diastolic function parameters for patients with recovery, compared to patients needing cardiac transplant or mechanical assist devices and patients with persistent abnormal LV systolic function and/or LV dimensions are shown in Table 3. All parameters studied, derived for mitral lateral and septal velocities on TDI, mitral valve inflow Doppler, isovolumic relaxation time, left atrial area z-score and lateral E/e’ ratio were not significantly different between the two groups.

## 4. Discussion

To the best of our knowledge, this study is the first to address the issue of diastolic dysfunction in paediatric dilated cardiomyopathy.

Clinical characteristics of the patients are in line with the literature with most patients having idiopathic dilated cardiomyopathy [15] and probable myocarditis being the most common cause leading to recovery [6]. Our results are also consistent with the literature on systolic function; lower values for LV diastolic diameter z-scores and better systolic function at initial assessment being associated with recovery [2,5].

However, in the current study, values for selected echocardiographic diastolic function parameters did not show significant association with outcome categories.

This may be explained by the fact that these parameters are highly influenced by age, heart rate, especially in young children, and load, which is a problem in children with dilated cardiomyopathy. In fact, the population studied had a wide age range. For obvious reasons, most patients with a progressive deterioration needed mechanical assist device implantation or heart transplant, which explains their shorter length follow-up.

Outcomes of paediatric patients diagnosed with dilated cardiomyopathy are bleak, as only about one third of these patients will ultimately have a complete recovery. Tools permitting a better risk stratification would be helpful in order to better target management of these patients. Indeed, it might be particularly helpful to be able to identify patients likely to recover, as opposed to patients needing closer follow-up, and who may have persistent left ventricular impairment or even progressive deterioration.

Several studies have focused on echocardiographic parameters as they are thought to provide a more objective assessment of disease severity, compared to clinical symptomatology.

A retrospective, single centre study by Mondal et al. [16] identified that an increase in the systolic to diastolic duration, an index of global ventricular function predicting both systolic and diastolic dysfunction, was associated with worse prognosis in patients with idiopathic, genetic or familial dilated cardiomyopathy. Through a prospective, multicentre study, including 127 patients with dilated cardiomyopathy, Molina et al. [5] presumed that diastolic function parameters of mitral inflow by Doppler and by tissue Doppler imaging were associated with disease progression, but these parameters could not be included as a definite result.

The current, pilot study exploited available data to try solving the question whether echocardiographic diastolic dysfunction parameters can predict dilated cardiomyopathy recovery. It presents at least two major strengths. The first is the serial echocardiography with standardized procedures and measures. The second is the respectable follow-up of >3.5 years.

On the other side, this study is subject to the usual limitations of a retrospective and single-centre study. Furthermore, this being an explorative, retrospective study, the small sample size available limits the power and interpretability of the results. Third, echocardiographic diastolic function parameters in Paediatrics have many limitations, and assessment of diastolic function needs the integration of different parameters. However, most parameters are simple measurements and easily feasible in clinical setting. Finally, this study focused on the initial echocardiographic findings, while the prognostic value of diastolic function may be greater in stable outpatients, presenting in regular follow-up clinics and having serial measurements.

## 5. Conclusions

This is the first study to explore the prognostic value of diastolic function in children presenting with dilated cardiomyopathy. Although the small sample does not allow a firm conclusion, selected echocardiographic measurements for diastolic function did not show a significant association with outcomes. Studies with bigger sample size, including stable outpatients presenting in heart failure clinics and involving several centres may be helpful and should be considered. Finally, it might be worth integrating novel diastolic parameters in a comprehensive diastolic assessment [17].

## Figures and Tables

**Table 1:** Clinical characteristics.

	Mechanical Assist Device/Cardiac Transplant	Stabilization	Recovery
Number assessed (%)	8 (18%)	24 (56%)	11 (26%)
Median age at assessment, y (range)	5.3 (0.79–13.2)	1.4 (0.52–3.8)	1.6 (1.0–3.7)
Median duration of follow-up, y (range) *	1.50 (0.23–2.83)	4.13 (1.44–7.20)	4.15 (3.54–7.51)
Weight (kg)	16.5 (9.2–41.9)	11.0 (6.8–14.8)	10.9 (8.8–14.7)
Height (cm)	125 (87–147)	90 (70–103)	91 (85–97)
Cause: -probable myocarditis, number (%)	1 (13%)	4 (17%)	6 (55%)
- Idiopathic	5 (62%)	16 (66%)	3 (27%)
- Familial	2 (25%)	4 (17%)	2 (18%)

* *p* = 0.0113 while comparing the 3 different groups. Dunn post-test showed a *p* = 0.012 while comparing mechanical assist device/cardiac transplant versus recovery and a *p* = 0.0033 versus stabilization. No other significant differences were detected while comparing the 3 different patients’ groups.

**Table 2:** Echocardiographic data for LV dimensions and systolic function. Data are presented as median and interquartile range (IQR).

	Mechanical Assist Device/Cardiac Transplant or Persistently Abnormal	Recovery	Number of Patients	*p*-Value
LVEDD z-score	7.9 (5.7–9.7)	5.0 (2.8–6.5)	43	0.0023
FS %	14.5 (8.3–19.0))	20.0 (11.8–23.0)	43	0.0363
Lateral S-wave velocity on TDI, m/s	0.04 (0.03–0.06)	0.050 (0.03–0.06)	43	0.7262
Septal S-wave velocity on TDI, m/s	0.05 (0.04–0.06)	0.060 (0.04–0.07)	43	0.6017

**Table 3:** Echocardiographic data for diastolic function. Data are presented as median and interquartile range (IQR).

	Mechanical Assist Device/Cardiac Transplant or Persistently Abnormal	Recovery	Number of Patients with Available Data for Analysis	*p*-Value
Mitral lateral E/e’ ratio	12.9 (8.8–20.2)	10.5 (8.4–14.9)	43	0.2424
Lateral e’-velocity on TDI, m/s	0.07 (0.04–0.14)	0.11 (0.065–0.13)	43	0.2099
Septal e’-velocity on TDI, m/s	0.065 (0.05–0.11)	0.095 (0.063–0.11)	43	0.1415
Lateral a’-velocity on TDI, m/s	0.04 (0.03–0.05)	0.05 (0.033–0.058)	43	0.1248
Septal a’-velocity on TDI, m/s	0.06 (0.05–0.07)	0.05 (0.043–0.06)	43	0.3096
IVRT, ms	60.0 (48.0–74.0)	66.5 (59.1–71.0)	34	0.7662
LA area z-score	3.4 (1.3–6.8)	3.8 (3.3–4.8)	25	0.6347
Mitral E-velocity, m/s	1.0 (0.80–1.1)	1.1 (0.90–1.3)	43	0.3019
Mitral A-velocity, m/s	0.60 (0.41–0.81)	0.68 (0.50–0.91)	38	0.3954
Mitral E-velocity deceleration time, ms	87.5 (74.8–113.8)	99.3 (89.3–108.8)	43	0.4076
Mitral E/A ratio	1.50 (1.20–2.40)	1.65 (1.10–1.93)	38	0.6524

## Data Availability

The data supporting this study are available from the corresponding author upon reasonable request.
